# Charting a novel path towards Ebola virus disease preparedness: Considerations for preventive vaccination

**DOI:** 10.1371/journal.pmed.1004543

**Published:** 2025-02-24

**Authors:** Donovan Guttieres, Charlot Diepvens, Rebecca F. Grais, Jackline Kiarie, Nico Vandaele, Catherine Decouttere

**Affiliations:** 1 Access-To-Medicines Research Centre, KU Leuven, Leuven, Belgium; 2 Pasteur Network, Paris, France; 3 Amref Health Africa, Nairobi, Kenya; Burnet Institute, AUSTRALIA

## Abstract

Donovan Guttieres and colleagues discuss the role of preventative vaccination in the fight against Ebola virus disease, as well as the various considerations that are required to ensure its success.

## Teaser

In June 2024, Gavi, the Vaccine Alliance, launched a preventive vaccination program against Ebola virus disease (EVD). This marks a historic shift in the management of EVD, allowing at-risk countries across sub-Saharan Africa to request support in implementing preventive vaccination campaigns. This perspective piece shares considerations that can inform how countries approach preventive EVD vaccination and potential unintended consequences. It also provides insights into strategies for vaccines against other epidemic-prone pathogens.

Largely neglected since its discovery in 1976, Ebola virus disease (EVD) received greater attention following the unprecedented 2014 to 2016 epidemic in West Africa. Since then, 9 EVD outbreaks caused by the Ebola virus (EBOV) have been reported [1]. To accelerate vaccine deployment and response efforts, a 500,000-dose global emergency stockpile was established in 2021 [2]. However, efforts to manage recent EVD outbreaks highlight challenges such as ineffective contact tracing and viral persistence in survivors, which increase the risk of new chains of transmission [3,4]. Such challenges point to the need for improved preventive strategies.

In June 2024, Gavi the Vaccine Alliance (formerly referred to as the Global Alliance for Vaccines and Immunization) launched the first program that supports preventive vaccination against EVD for 18 East, West, and Central African countries [5]. These countries have previously reported confirmed cases of EVD (including imported cases) or share a border with a country that has experienced an outbreak resulting from a suspected animal-to-human spillover event. The program includes funding for vaccine procurement, with no co-financing requirement, as well as cash grants and technical assistance to support planning, implementation, monitoring, and evaluation of activities.

The launch of a preventive EVD vaccination program follows updated recommendations from the Strategic Advisory Group of Experts on Immunization (SAGE) on the use of 2 vaccines, prequalified by the World Health Organization (WHO), against EBOV [6]. SAGE recommends proactive vaccination in priority areas and target populations, such as healthcare (e.g., medical personnel, traditional healers) and frontline (e.g., taxi drivers) workers, and to a lesser extent EVD survivors and their contacts if doses are available. Previously, vaccines were only used reactively during outbreaks. One exception has been re-purposing doses approaching expiry in the global stockpile for use in the absence of outbreaks, including in the Democratic Republic of the Congo (DRC), Guinea-Bissau, Uganda, and Sierra Leone [7]. The initial focus on vaccination in outbreak settings was partly due to limited evidence on effectiveness beyond the “Ebola Ça Suffit” trial conducted in 2015, as well as supply constraints at the time. Since then, the supply situation has improved and evidence of the protective effect of EVD vaccines has accumulated [8].

To benefit from the program, eligible countries are expected to conduct a situational analysis to understand restrictive and supportive factors surrounding preventive EVD vaccination among target populations and use these findings to request Gavi support. Countries also need to present how their proposed EVD vaccination plan complements other EVD-related efforts and immunization plans, as well as share advocacy, risk communication, and community engagement strategies to overcome barriers to access. However, countries most at risk of EVD have faced multiple concurrent emergencies, such as Mpox and cholera outbreaks, gaps in routine immunization, extreme weather events due to climate change, and humanitarian crises. Constrained budgets, high levels of debt servicing relative to health expenditure, and over-reliance on development aid have further pushed governments to focus on other competing priorities. This has affected efforts to sustain EVD preparedness, which are often characterized by cycles of panic and neglect. A sporadic response only when disease threats materialize exacerbates mistrust among communities. Therefore, even as Gavi’s preventive EVD vaccination program has launched, assessing country-level demand and associated programmatic needs is not trivial.

The value of preventive EVD vaccination depends on numerous time-dependent dynamics. Given the uncertainty around when and where future EVD outbreaks might emerge, the timing of a country’s first preventive vaccination campaign is crucial. Dependencies between decisions (e.g., country applications, Gavi approval, campaign planning) and context-specific operational challenges can quickly accumulate, delaying implementation and leaving populations vulnerable.

Preventive EVD vaccination may also encounter community resistance if resources are used in the absence of an immediate threat from outbreaks. Furthermore, perceptions around the value of preventive EVD vaccination could be challenged by previously mentioned (and other) competing priorities. Additionally, waning vaccine-induced immunity gradually reverses preparedness efforts. Despite increased evidence of correlates of vaccine-induced protection, no correlate has been formally validated in humans. Long-term durability is thus not well known for either of the 2 WHO prequalified vaccines. Therefore, continued research is needed to inform vaccine interchangeability, identify who might benefit most from re-vaccination, and when boosters may be needed to ensure sustained protection against EVD [9].

Defining who to vaccinate is also critical in amplifying the value of EVD vaccination, especially as the zoonotic niche of EBOV is projected to expand across sub-Saharan Africa [10]. Surveillance helps identify populations most at risk of EVD, for example, by detecting viral exposure and monitoring levels of immune protection within communities. Additionally, threat assessments based on a WHO framework for vaccination in acute humanitarian emergencies provide a basis for equitable access to vaccines, especially if supply is limited [11]. These assessments involve monitoring local epidemiological risks of EVD and contextual constraints to inform timely decisions.

As countries consider the implementation of preventive EVD vaccination, the vaccination strategies they put in place will need to align with predefined goals. For example, countries may look to minimize disease burden as outbreak risks evolve or minimize the cost per case averted. These different goals may lead to tradeoffs across public health outcomes and programmatic feasibility. Although higher vaccination coverage offers greater protection and promotes broader access, it may be too expensive and burden existing health systems. Models that integrate both vaccine demand and supply considerations can provide insights into such tradeoffs and unintended consequences across different goals, thus helping inform country-level EVD vaccination strategies [12]. At a global level, they also point to the importance of alignment across stakeholders, including governments, procurement agencies, and manufacturers. For example, increased vaccine demand can enhance the sustainability of the EVD supply market by stimulating higher volume production and lower prices. On the other hand, limited demand could point to commercial risks and reduced investments by manufacturers, jeopardizing timely vaccine availability.

Paving the way to healthy markets requires stakeholder coordination. This includes translating global SAGE recommendations into clear guidance on the use of vaccines at different levels, for example, through relevant regional and national advisory groups. Additionally, there are ongoing efforts to raise awareness of Gavi’s progam and support countries in preparing applications. These interactions also contribute to the development of strategic demand scenarios that will inform upcoming UNICEF (formerly referred to as the United Nations International Children’s Emergency Fund) tenders for the procurement of EVD vaccines. These tenders are critical to ensure long-term demand guarantees that incentivize manufacturers and enable production planning. This may also involve transition of the global stockpile from a static to revolving mechanism that can be designed to fulfill both outbreak and preventive demand.

Introducing new preparedness strategies should be needs-based and community-driven. For effective EVD prevention and control, local ownership of vaccination strategies needs to be complemented with government accountability on the use of vaccines and other resources once available. Additionally, sustained efforts are needed to realize aspirations outlined in International Health Regulations and to promote continuity of preventive measures, including knowledge sharing, learning, and collaboration across countries.

Investing in preventive vaccination also has the potential to strengthen health systems, allowing countries to better manage EVD and make resources available for other health priorities. Experience using EVD vaccines in non-outbreak settings can contribute to learnings around supply chain design. For example, ultra-cold chain requirements (−80°C to −60°C) for the storage of EVD vaccines allowed Ebola-affected countries to build capabilities useful in the distribution of mRNA vaccines during the COVID-19 pandemic. These capacities can also support the future introduction of vaccines with complex cold chain requirements, although it is essential to work towards solutions without the need for this infrastructure. Additionally, the focus of preventive EVD vaccination efforts among healthcare and frontline workers, as well as EVD survivors and their contacts, highlights the importance of outreach strategies focused on adult populations, compared to those focused only on children. Therefore, the implementation of preventive EVD vaccination can reveal contextually relevant drivers of uptake, leading to more tailored communication and community engagement practices.

Finally, integrating preventive vaccination in multi-sectoral EVD management plans can foster resilience to better cope with unanticipated needs. Marking 10 years since the epidemic in West Africa, Gavi’s program charts a promising path toward the management and prevention of EVD outbreaks. Time will tell if and how it changes the course of EVD and the extent to which it provides a viable model for other vaccines against epidemic-prone pathogens.

## References

[1] US CDC. Ebola Disease Outbreaks by Species and Size, Since 1976. Mar 2023. Available from: https://www.cdc.gov/vhf/ebola/history/distribution-map.html.

[2] Kallay R, Doshi RH, Muhoza P, Choi MJ, Legand A, Aberle-Grasse E, et al. Morbidity and Mortality Weekly Report Use of Ebola Vaccines-Worldwide, 2021–2023. 2018. Available from: https://www.cdc.gov/mmwr/mmwr_continuingEducation.html. 10.15585/mmwr.mm7316a1 PMC11065462 38662631

[3] Keita M, Cherif IS, Polonsky JA, Boland ST, Kandako Y, Cherif MS, et al. Factors Associated with Reliable Contact Tracing During the 2021 Ebola Virus Disease Outbreak in Guinea. J Epidemiol Glob Health. 2024. doi: 10.1007/s44197-024-00202-y 38372893 PMC11442408

[4] Keita AK, Koundouno FR, Faye M, Düx A, Hinzmann J, Diallo H, et al. Resurgence of Ebola virus in 2021 in Guinea suggests a new paradigm for outbreaks. Nature. 2021;597: 539–543. doi: 10.1038/s41586-021-03901-9 34526718

[5] Vaccine Funding Guidelines (June 2024). In: Gavi, the Vaccine Alliance [Internet]. Jun 2024 [cited 2024 Dec 1]. Available from: https://www.gavi.org/sites/default/files/support/guidelines-2024/GAVI-Vaccine-Funding-Guidelines-aug2024.pdf.

[6] World Health Organization. Extraordinary meeting of the Strategic Advisory Group of Experts on Immunization on Ebola vaccination, May 2024: conclusions and recommendations. Weekly Epidemiological Record (WER). 2024;99:355–362. Available from: https://iris.who.int/bitstream/handle/10665/378110/WER9927-eng-fre.pdf?sequence=1.

[7] WHO. Ebola vaccine dashboard. 2024 [cited 2024 Dec 1]. Available from: https://www.who.int/groups/icg/ebola-virus-disease.

[8] Meakin S, Nsio J, Camacho A, Kitenge R, Coulborn RM, Gignoux E, et al. Effectiveness of rVSV-ZEBOV vaccination during the 2018–20 Ebola virus disease epidemic in the Democratic Republic of the Congo: a retrospective test-negative study. Lancet Infect Dis. 2024. doi: 10.1016/S1473-3099(24)00419-5 39178866

[9] Adriaensen W, Oostvogels S, Levy Y, Leigh B, Kavunga-Membo H, Watson-Jones D. Urgent considerations for booster vaccination strategies against Ebola virus disease. Lancet Infect Dis. 2024. doi: 10.1016/S1473-3099(24)00210-X 38734010

[10] Redding DW, Atkinson PM, Cunningham AA, Lo Iacono G, Moses LM, Wood JLN, et al. Impacts of environmental and socio-economic factors on emergence and epidemic potential of Ebola in Africa. Nat Commun. 2019;10. doi: 10.1038/s41467-019-12499-6 31615986 PMC6794280

[11] Vaccination in acute humanitarian emergencies: a framework for decision making. Geneva; 2017. Available from: https://www.who.int/publications/i/item/WHO-IVB-17.03.

[12] Guttieres D, Diepvens C, Decouttere C, Vandaele N. Modeling Supply and Demand Dynamics of Vaccines against Epidemic-Prone Pathogens: Case Study of Ebola Virus Disease. Vaccines (Basel). 2024;12. doi: 10.3390/vaccines12010024 38250837 PMC10819028

